# Short-Term Effects of a Novel Eye Mask Producing Heat and Vibration for the Treatment of Meibomian Gland Dysfunction: A Pilot Study

**DOI:** 10.1155/2021/1370002

**Published:** 2021-08-17

**Authors:** Luca Vigo, Marco Pellegrini, Francesco Carones, Vincenzo Scorcia, Giuseppe Giannaccare

**Affiliations:** ^1^Carones Ophthalmology Center, Milan, Italy; ^2^Department of Ophthalmology, Ospedali Privati Forlì “Villa Igea”, Forlì, Italy; ^3^Istituto Internazionale per La Ricerca e Formazione in Oftalmologia (IRFO), Forlì, Italy; ^4^Department of Ophthalmology, University Magna Græcia of Catanzaro, Catanzaro, Italy

## Abstract

**Purpose:**

To investigate the short-term effects on tear film parameters and ocular symptoms of a novel eye mask producing heat and vibration developed for the treatment of dry eye disease owing to meibomian gland dysfunction (MGD).

**Methods:**

This is a pilot study including the first 20 consecutive patients (6 males, 14 females; mean age 52.4 ± 16.8 years) who were treated with a novel eye mask producing heat (42°) and vibration (up to 20 Hz) (Activa, SBS Sistemi, Turin, Italy) for 15 minutes. The treatment incorporates 2 phases in the following chronological order: 5 minutes of heating (phase I); 10 minutes of combination of heating and vibration (phase II). Noninvasive ocular surface examination was carried out before (T0) and 30 minutes after the mask session (T1) by means of Idra (SBS Sistemi, Turin, Italy) for the measurement of noninvasive break-up time (NIBUT) and lipid layer thickness (LLT). Patients' satisfaction after treatment was ascertained by asking the patients whether they perceived improvement from their baseline symptoms according to a 5-grade scale: none = 0; trace = 1; mild = 2; moderate = 3; high = 4.

**Results:**

All patients completed regularly the mask session and no device-related adverse events were noted. NIBUT improved significantly from T0 to T1 (from 7.2 ± 1.8 s to 8.1 ± 2.1; *P* = 0.014). In parallel, also LLT improved from from T0 to T1 (72.5 ± 13.9 nm to 83.1 ± 16.1; *P* = 0.016). Seven patients (35% of the total) had a moderate satisfaction (grade 3) while 13 patients (65%) had a high satisfaction (grade 4) with treatment.

**Conclusions:**

This eye mask represents a novel well tolerated tool in the armamentarium of MGD treatments. Thirty minutes after the session, NIBUT and LLT increased significantly; furthermore, all patients reported an improvement of discomfort symptoms with a moderate to high satisfaction with treatment.

## 1. Introduction

Meibomian gland dysfunction (MGD) is the most common cause of evaporative dry eye disease (DED) that represents the main subtype of ocular surface disease [1–3]. The aim of MGD treatment is to increase the flow of meibum secretion thus restoring the release of the lipid layer of the tear film [4–6]. Apart from medications, eyelid hygiene is the main conservative treatment and involves the use of warm compresses for the local application of heat followed by self-administered mechanical massage of the lid margin [7]. However, it is difficult for the patient to maintain a standardized and reproducible eyelid hygiene. The main obstacle is represented by the need for reheating every 2 minutes the compresses in order to maintain the therapeutic levels of heat. Furthermore, the mechanical force exerted on the eyelids varies considerably depending on both method employed and patient [8]. Moreover, patient compliance is an additional limitation to eyelid hygiene, which conversely should require faithful regularity (10 minutes every morning and evening) for achieving best results [9].

In order to overcome these drawbacks, novel devices specifically designed for in-office MGD management, able to produce (i) heating, (ii) heating plus humidity, or (iii) heating and massaging, are emerging in the market [7]. Among these, a novel eye mask producing therapeutic heat (42°) and vibration (up to 20 Hz) for 15 minutes has been recently developed (Activa, SBS Sistemi, Turin, Italy). The aim of the present pilot study is to provide preliminary results about the short-term effects on tear film parameters and subjective symptoms of this novel eye mask used in patients in MGD.

## 2. Materials and Methods

### 2.1. Study and Patients

This pilot study included consecutive MGD patients who attended for a routine visit to the ocular surface office of the University Hospital of Catanzaro (Italy) between February 2021 and March 2021. Patients aged 19–80 years were screened for eligibility according to the following inclusion criteria: presence of MGD defined by the presence of signs consistent with meibomian gland terminal duct obstruction with abnormal quantity and/or quality of meibomian gland secretions; presence of at least one MGD-related ocular symptom such as dryness, foreign body sensation, irritation, and burning; pathological value of Ocular Surface Disease Index (OSDI) score (≥13); and noninvasive break-up time (NIBUT) < 10 s. Patients were excluded from the study if one of the following conditions was present: active eye inflammation; eyelid malposition such as entropion, ectropion, and ptosis; recent ocular surgery (within 3 months); history of contact lens wearing; risk of retinal detachment such as high myopia, lattice degeneration, and retinal break; and usage of anti-inflammatory eye drops (topical steroid or cyclosporine) within 1 month.

The study followed the tenets of the Declaration of Helsinki for research involving human subjects. Written informed consent was obtained from all participants after the nature and possible consequences of the study had been explained to them.

### 2.2. Treatment and Diagnostic Tools

**Figure 1:**
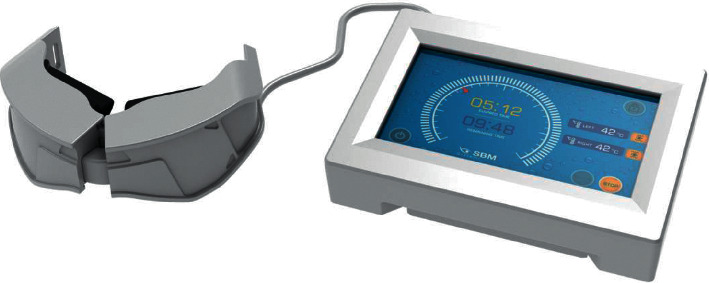
The eye mask Activa (SBS Sistemi, Turin, Italy).

Patients were treated with a single session of a newly-developed eye mask (Activa, SBS Sistemi, Turin, Italy) (Figure 1). Briefly, through a fully automated procedure (touch-screen controlled and user-friendly), the device is able to melt the meibum inside the glands and simultaneously squeeze them. The entire treatment is 15 minutes long and incorporates 2 phases in the following chronological order: 5 minutes of heating at 42°C (phase I); 10 minutes of combination of heating at 42°C and vibration up to 20 Hz (phase II). All patients were examined before starting the session (T0) and 30 minutes after the end of the session (T1) by means of Idra (SBS Sistemi, Turin, Italy) for the measurement of NIBUT and lipid layer thickness (LLT) [10]. Treatment tolerability and patients' satisfaction with treatment were ascertained at T1 by asking the patients whether they perceived improvement from their baseline discomfort symptoms according to a 5-grade scale: none = 0; trace = 1; mild = 2; moderate = 3; high = 4.

### 2.3. Outcome Measures

The primary outcome measures were improvement of NIBUT after the mask session (T1) as well as tolerability of the procedure (5-grade questionnaire). Secondary outcome measure included change of LLT after the mask session (T1).

### 2.4. Sample Size

To determine the required sample size, we performed a power analysis based on the mean difference in break-up time reported in a previous study employing another thermal massager [11]. With this assumption, a samples size of 12 patients would yield a power of 95% with a paired sampled *t*-test. However, to ensure adequate reliability, we aimed for a sample size of 20 patients.

### 2.5. Statistical Analysis

Patients received treatments in both eyes. Data for statistical analysis were derived from the eye with the lowest NIBUT value (study eye). Statistical analysis was performed using R (version 4.0.0) and RStudio (version 1.2.5042) software. The Wilcoxon test was used to compare the change of NIBUT and LLT before (T0) and after the mask session (T1). A *P* value of less than 0.05 was considered statistically significant.

## 3. Results

The first 20 consecutive MGD patients (6 males, 14 females, mean age 52.4 ± 16.8 years) who fulfilled the inclusion/exclusion criteria were enrolled in this pilot study. Mean OSDI score at baseline was 23.5 ± 21.1. Meibomian gland dysfunction was associated with rosacea in 3 patients (15%), atopic dermatitis in 2 patients (10%), and Sjögren syndrome in 2 patients (10%). In the remaining 13 patients (65%), MGD was present as an isolate ocular disorder without any systemic disease linked to its occurrence. Previous treatments used by patients for controlling MGD included warm compresses and self-administered eyelid massage in 12 patients (60%), topical tetracycline ointment in 4 patients (40%), and systemic doxycycline in 2 patients (20%).

**Figure 2:**
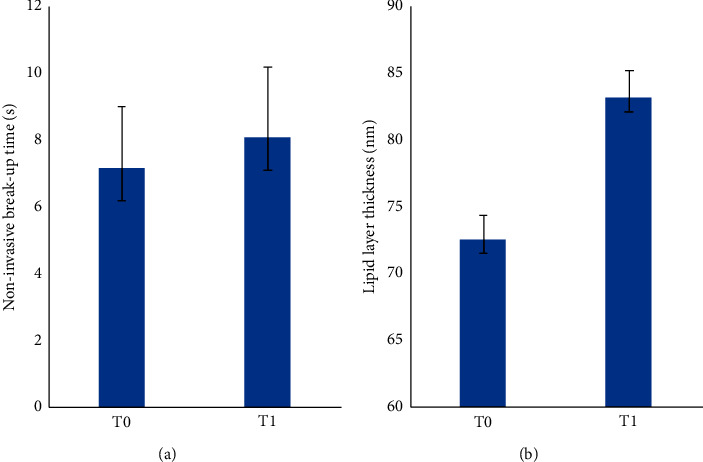
Histograms showing noninvasive break-up time (a) and lipid layer thickness (b) before and after the mask session.

All patients completed regularly the entire mask session. No device-related adverse events were noted. NIBUT improved significantly from T0 to T1 (from 7.2 ± 1.8 s to 8.1 ± 2.1; *P* = 0.014) (Figure 2(a)). In parallel, also LLT improved from T0 to T1 (72.5 ± 13.9 nm to 83.1 ± 16.1; *P* = 0.016) (Figure 2(b)). Seven patients (35%) had a moderate satisfaction (grade 3) while 13 patients (65%) had a high satisfaction (grade 4) with treatment.

## 4. Discussion

New medical and physical therapies for MGD management are continuously becoming commercially available, aiming at helping clinicians and patients to better cope with the disease [2, 12–16]. Warm compresses and self-administered eyelid massage represent the main treatments used for MGD. Unfortunately, the efficacy of this approach is often disappointing due to various reasons. Firstly, traditional warm compresses do not stay hot for a sufficient amount of time, requiring frequent reheating, which makes their administration a labor-intensive and time-consuming daily treatment, thus reducing patients' compliance [9]. Secondly, the mechanical force exerted on the eyelids varies considerably according to the method used [8]. Controlled devices, in the form of eye mask or goggle, can potentially standardize the application of heat treatment, controlling the temperature throughout the entire treatment and maintaining it at a therapeutic level. Furthermore, some of these devices are able to combine heating with a gentle and constant vibration, allowing the lipids to be massaged out of the meibomian glands [8, 11].

The mechanism by which the thermal massage improves the symptoms of DED is thought to be multifactorial. Firstly, vibration determines a mechanical pressure able to deliver to the ocular surface the meibum liquefied by heating. However, also other mechanisms have been proposed. Similar to acupuncture, vibration and massage may reduce chronic inflammation of the ocular surface through the cholinergic anti-inflammatory pathway, by enhancing vagus nerve activity [17]. In addition, ocular irritation, one of the symptoms of MGD, can be decreased through the analgesic effects of thermotherapy. Heat also acts selectively on tissue and free nerve endings, directly or indirectly reducing pain [18, 19].

Different types of devices have been developed in the recent past for the treatment of obstructive MGD, acting by heating with or without massaging. Among these, the LipiFlow system (Johnson & Johnson Vision, Jacksonville, FL, USA) is the most studied one. A recent meta-analysis found that vectored thermal pulsation therapy is highly effective at restoring meibomian gland function and also reducing DED symptoms. In addition, it is effective in improving other downstream correlates of ocular surface health such as TBUT, LLT, ocular surface staining, and, in some cases, tear osmolarity [19]. A recent clinical trial found that iLUX system (Alcon, Fort Worth, TX, USA) provides outcomes clinically equivalent to those of the LipiFlow [20]. Another thermal massager (Nurieye-5800, Seodong Medical, Busan, Republic of Korea) has been shown to provide better symptomatic relief in MGD patients compared to 0.1% sodium hyaluronate-based tear substitutes, but data about changes of LLT after treatment were not investigated [11].

In the present study, a newly-developed eye mask producing heating in the first phase of session and heating plus vibration in the second phase has been used for the treatment of patients with symptomatic MGD. Since MGD may be associated with other systemic diseases such as rosacea and Sjogren syndrome [21, 22], a heterogeneous MGD population was enrolled to obtain results that are generalizable to the real-life clinical practice. This preliminary analysis showed that half hour after the first mask session, tear film parameters (NIBUT and LLT) improved significantly. In parallel, all patients reported moderate to high satisfaction with treatment, describing a good ocular comfort during and after the procedure.

Compared to LipiFlow, the device leader in the MGD market, the present eye mask has some potential advantages considering that it is less invasive and cheaper (the cost is about 6–7.000 €) and a version for home-use is available for patients.

Our pilot study suffers from several limitations that deserve mentioning. Among these, the main limitation concerns study design as we measured the short-term effects of a single eye mask session on tear film parameters and subjective symptoms. However, a prospective controlled study with larger cohort, longer follow-up, and a more comprehensive ocular surface workout is currently running at our institution in order to provide a scientific validation of this device by analyzing more in depth the benefits of this new automated treatment modality for MGD.

## 5. Conclusions

This innovative eye mask combines both heating and automatic squeezing technology in a fast and painless procedure. All the objective parameters studied improved significantly after the mask session. The device had also an excellent safety and tolerability profile since patients reported a distinct improvement in ocular comfort and a significant reduction of their MGD symptoms.

## Data Availability

The datasets generated during and/or analysed during the current study are available from the corresponding author on reasonable request.
